
JOURNAL INFORMATION
==============================
NLM Title Abbreviation: Inter Econ
Journal ID: Inter Econ
NLM Journal ID: 101086399

Inter Economics

ISSN: 0020-5346

ARTICLE INFORMATION
==============================
PMCID: PMC8176661
PMID: 34103757
DOI: 10.1007/s10272-021-0966-9
Article ID: 966
Article version: 1
Subjects: Editorial

Europe’s Vaccine Paradox: From Supply to Demand Issues

Bongardt Annette abongardt@uevora.pt
1
Torres Francisco ftorres@ucp.pt
2
1 grid.8389.a 0000 0000 9310 6111 Palácio do Vimioso, CICP - University of Évora, Largo Marquês de Marialva 8, AP 94, 7002-554 Évora, Portugal
2 grid.7831.d 000000010410653X Católica Lisbon School of Business and Economics, Palma de Cima, 1649-023 Lisboa, Portugal
Electronic publication date: 2021 Jun 4
Print publication date: 2021
Volume: 56
Issue: 3
First page: 130
Last page: 131
Copyright: © The Author(s) 2021
License: Open Access: This article is distributed under the terms of the Creative Commons Attribution 4.0 International License (https://creativecommons.org/licenses/by/4.0/).
Open Access funding provided by ZBW — Leibniz Information Centre for Economics.
License URL: https://creativecommons.org/licenses/by/4.0/

issue-copyright-statement: © The Author(s) 2021

==============================

The collective action on vaccine procurement has attracted much criticism, but not necessarily for the right reasons. It successfully protected the internal market and EU values from vaccine nationalism. BioNTech became a European success story, and EU vaccine deliveries and vaccination are gaining speed. Yet, the demand side to vaccines brings the role of the European sectoral regulator to the forefront.

Public health is one of those policy areas in which competences remained almost exclusively with EU member states. With the exception of common safety concerns, including protection of the internal market, EU actions in public health are supportive in nature (as is the case of the EU's health strategy that complements member states' national health services and medical care).

The EU vaccine strategy was set up as a joint action at the EU level, agreed upon by the Commission and the member states, when uncoordinated member state responses to the COVID-19 crisis had repeatedly put common goods (public health, but also the internal market and Schengen) and European values (solidarity) at risk. The Commission took over the vaccine procurement initiative for the entire EU to prevent wasteful competition for scarce vaccines between member states and protect smaller ones from being charged higher prices or even losing out. A group of member states (France, Germany, Italy and the Netherlands) had started to negotiate supplies of large quantities of a given vaccine (Oxford-AstraZeneca) just for themselves in the summer of 2020. Joint procurement would protect the internal market (and with it European integration) from potentially disruptive effects of member states fending for themselves and make use of the bloc's bargaining power for the benefit of all member states in an industry characterised by a limited number of large suppliers and worldwide competition for vaccines.

The fact that the European Commission negotiated vaccines for the member states, drawing on its expertise in trade negotiations, resulted in very competitive prices internationally (as an accidentally leaked price list well illustrates). That said, the European Commission has admitted to shortcomings stemming from a lack of negotiation experience with the specific sector (pharmaceutical industry), which led it to initially underestimate supply issues and the need for production capacity building; those issues were subsequently addressed.

The EU's vaccine portfolio represents the possible common denominator. It reflects that member states harbour different preferences for national suppliers and production, the type of vaccines, prices, etc. (which explains why BioNTech-Pfizer's initial offer to supply very large quantities of its novel messenger ribonucleic acid (mRNA) vaccine was scaled down) and constraints (financial). The Commission entered advance purchase agreements with a set of six potential suppliers and two different types of vaccines (vector-based and mRNA) with the consent of all member states. Member states were entitled to buy a specified number of vaccine doses in a given time period and at given prices for their domestic vaccination campaigns and were free to decide not to make use of their pro-rata share (proportional to their population compared to the EU's).

Regrettably, this built-in flexibility has not prevented countries that have opted to buy less than their share of available vaccines to rather unethically and opportunistically blame the Commission for their own choices when AstraZeneca failed to honour its delivery commitments and to accuse other member states of failing them on solidarity grounds. The EU, a rules-based organisation and staunch defender of multilateralism, stayed true to its values. It did not succumb to European vaccine nationalism even in a situation of relative vaccine scarcity, standing out as a major exporter of vaccines and contributor to COVAX.

Since the second quarter of 2021, EU vaccine deliveries and vaccination have been gaining speed thanks in part to increasing production capacity in the EU and anticipated additional deliveries from BioNTech-Pfizer. Another 1.8 billion doses of its vaccine have been secured for 2022-23.

While the collaborative action seems on track with regard to supply side issues, a demand issue arose. Vaccines are a pharmaceutical good, requiring regulatory approval. This takes time but is necessary to ensure public trust, which is essential. As vaccination is voluntary, safety and efficacy is key for the acceptance and uptake of the vaccine and the success of vaccination campaigns. The vaccines that were contracted and granted European Medicines Agency (EMA) approval not only have different cost-effectiveness properties but also vary in their efficacy against the original virus and against mutants, in the ease in updating it and, not least, in side effects.

In short, they are not a homogeneous product, which leaves the EU in a paradoxical situation. The mRNA vaccine by BioNTech-Pfizer is a European success story and at the basis of the successful early vaccination campaigns in the US and in Israel (and also the start of the UK campaign). Yet, those countries took risks that the EU, with a comparatively vaccine-cautious population, arguably could not. It is doubtful that Europeans would have been prepared to go along with emergency approvals and testing vaccines first, even less so vaccines with a novel approach untested on a large scale. It was after having witnessed the high efficacy and milder side effects that Europeans warmed to mRNA vaccines.

The rollout of mRNA (BioNTech-Pfizer and Moderna) and vector-based vaccines (AstraZeneca, later also Johnson & Johnson) introduced "competition in the market" in the EU. Consumers/citizens will make a rational choice between available vaccines, from an individual (not societal) perspective on the basis of what they consider trustworthy information. The choice before them was between mRNA vaccines, increasingly established as the vaccine "gold standard", and AstraZeneca's vaccine, beset by problems from the beginning (data issues, delivery failures, serious collateral effects). After insisting on nevertheless going ahead with the AstraZeneca vaccine, most EU countries have subsequently suspended its use (below a certain age, or even for all age groups) in light of rare but serious side effects.

The handling of the AstraZeneca case by the European regulator was hardly liable to build trust. EMA, which had granted AstraZeneca's vaccine conditional approval (which the US health authorities have not to date), did not pause it to analyse reported cases of blood clots, unlike many other national regulators (and the American Centers for Disease Control and Prevention and Food and Drug Administration in the case of the Johnson & Johnson vaccine). Instead, EMA's insistence that the vaccine was safe and that the benefit-risk profile was positive echoed that of the British Medicine and Healthcare products Regulatory Agency, although the latter eventually recommended age group limitations. EU national health authorities stepped in to analyse causality, risks, etc., and ended up deciding nationally on restrictions to the use of the vaccine.

The Commission has drawn the logical conclusion: It did not prolong the contract with AstraZeneca and will go with 'proven reliable' suppliers with EU production capacity in the future. In the meantime, while the US will give away mostly vector-based vaccines, in the EU politicians and some health authorities insist that vaccines that happen to be in stock are good enough to get faster (possibly illusionary) herd immunity. Yet that may erode the trust of citizens concerned that societal benefits may come at an individual cost. At the end of the day, defeating COVID-19 calls for a strategic and longer-term approach on vaccines – a high level of protection, also against new variants, an ease of adaptation combined with reliable supplies – but requires a high uptake, which rests on maintaining citizens' trust in European institutions. Citizens should thus not be denied the right to choose when there is a portfolio of vaccines.

Annette Bongardt, CICP — University of Évora; and Universidade Fernando Pessoa, Porto, Portugal.

Francisco Torres, Católica Lisbon School of Business and Economics, Portugal.

